# Dissecting the causal pathway linking HbA1c to oral cavity cancer via immune cell modulation: a bidirectional mendelian randomization analysis

**DOI:** 10.3389/fonc.2026.1768761

**Published:** 2026-02-13

**Authors:** Jieqi Wang, Zhe Zhang, Yujia Wang, Bowen Li, Meihua Zheng, Jinsong Li

**Affiliations:** 1 Department of Oral and Maxillofacial Surgery, Department of General Dentistry, Sun Yat-sen Memorial Hospital, Sun Yat-sen University, Guangzhou, Guangdong, China; 2 The First People’s Hospital of Foshan (Foshan Hospital Affiliated to Southern University of Science and Technology), School of Medicine, Southern University of Science and Technology, Guangdong, China

**Keywords:** glycated hemoglobin, immune-mediated, Mendelian randomization, oral cavity cancer, tumor microenvironment

## Abstract

**Objective:**

To investigate the immune-mediated mechanisms underlying the causal link between HbA1c and oral cavity cancer.

**Methods:**

A bidirectional two-sample Mendelian randomization (MR) framework was employed, utilizing genome-wide association study (GWAS) data on HbA1c (n=46,368), oral cavity cancer (357 cases/372,016 controls), and 731 immune cell traits. The primary IVW analysis was supported by sensitivity analyses, including Cochran’s Q, I², MR-Egger, and leave-one-out, to ensure robustness. A multivariable MR (MVMR) was implemented to investigate the mediating effect of immune cells within the causal pathway linking HbA1c and oral cavity cancer. *In vitro* experiments evaluated the impact of advanced glycation end products (AGEs) on oral squamous cell carcinoma (OSCC) viability and CD8+ T cell function.

**Results:**

Genetically elevated HbA1c was causally associated with the risk of oral cavity cancer (OR = 0.9993, 95% CI: 0.9988–0.9997, P = 0.0021). HbA1c significantly influences the activity of activated B cells and T cells (*P* < 0.05). MVMR analysis showcased considerable variation across immune cell types in relation to the HbA1c-oral cavity cancer relationship (*P* < 0.05). Importantly, reverse MR analyses revealed no significant bidirectional association between oral cavity cancer and HbA1c (OR = 0.1591; *P* = 0.1732). *In vitro*, AGEs did not alter OSCC cell viability but suppressed CD8+ T cell production of IFN-γ and GZMB, increased oxidative stress, and reduced tumor cell susceptibility to T-cell cytotoxicity (all P<0.05).

**Conclusion:**

This study provides genetic and experimental evidence for a protective, immune-mediated role of HbA1c in oral cavity cancer. While AGEs do not directly impact OSCC viability, they impair CD8+ T cell function via oxidative stress, highlighting the interplay between glycemic control, immune modulation, and carcinogenesis.

## 1 Introduction

Head and neck carcinoma represents the sixth most common cancer worldwide and is a significant contributor to global cancer morbidity and mortality (1). Oral cavity cancer is the predominant subtype of head and neck carcinomas, with a multifactorial etiology that includes tobacco use, alcohol and betel nut consumption, genetic susceptibility, poor oral hygiene, and human papillomavirus infection. The major clinical treatments vary according to disease stage, including surgical resection, radiotherapy, chemotherapy and immunotherapy (2). Despite ongoing advancements in therapies and increased recognition of the underlying causes, survival rates for oral cavity cancers have improved only marginally in recent decades (3). This emphasizes the pressing requirement for pioneering biomarkers and therapeutic targets that could improve patient outcomes.

As a clinical biomarker, HbA1c is characterized by its ability to integrate average glycemic exposure over approximately three months, independent of fasting status. Furthermore, this test demonstrates excellent consistency and is unaffected by daily fluctuations in glucose levels (4). HbA1c has exhibited superior predictive capabilities for the risk of vascular and cardiometabolic diseases, even in individuals without diabetes (5). Hyperglycemia has been implicated in the pathogenesis and unfavorable prognosis of oral cavity cancer (6–9). However, other studies demonstrated that there was no direct correlation between diabetes and oral cavity cancer (10, 11). The observed inconsistency may reflect methodological constraints inherent in observational designs, most notably confounding and reverse causality. Evaluating the relationship between HbA1c and oral cavity cancer is difficult due to the complex interactions of several clinical and social factors, such as smoking, alcohol use, obesity, the duration of diabetes, and the use of hypoglycemic agents. The role of hyperglycemia in cancer pathogenesis remains unclear. A comprehensive understanding of the correlation between HbA1c and oral cavity cancer might unveil novel approaches for prevention and treatment.

Mendelian randomization (MR) is an analytical approach that uses genetic variants to infer causality while helping mitigate confounding. The MR analysis technique mitigates the influence of confounding variables by employing genetic variations present at birth, which are identified through genomic association studies, as instrumental variables for a particular exposure.

Beyond establishing a primary association, this study specifically investigates the immune-mediated mechanisms linking glycemic control to oral cavity carcinogenesis. Immune cells are known to play multifaceted roles in tumor progression and evasion of immune surveillance, while alterations in glycemic control might impact these cellular dynamics, potentially influencing susceptibility to and progression of cancer (12). Thus, the findings of this study contribute to our understanding of oral cavity cancer etiology and offer broader insights into the metabolic dysregulation underlying cancer biology.

Within an MR framework, we aim to evaluate the causal effects of HbA1c and immune cells on oral cavity cancer, a relationship less clear than those reported for other malignancies (5, 13, 14). The causal role of HbA1c in oral cavity cancer and the potential mediation by immune cells remain unexplored. Our study addresses this gap by performing the first Mendelian randomization analysis to elucidate this relationship and its immune-mediated pathways, thereby providing novel insights into HbA1c as a risk biomarker.

## 2 Materials and methods

**Figure 1:**
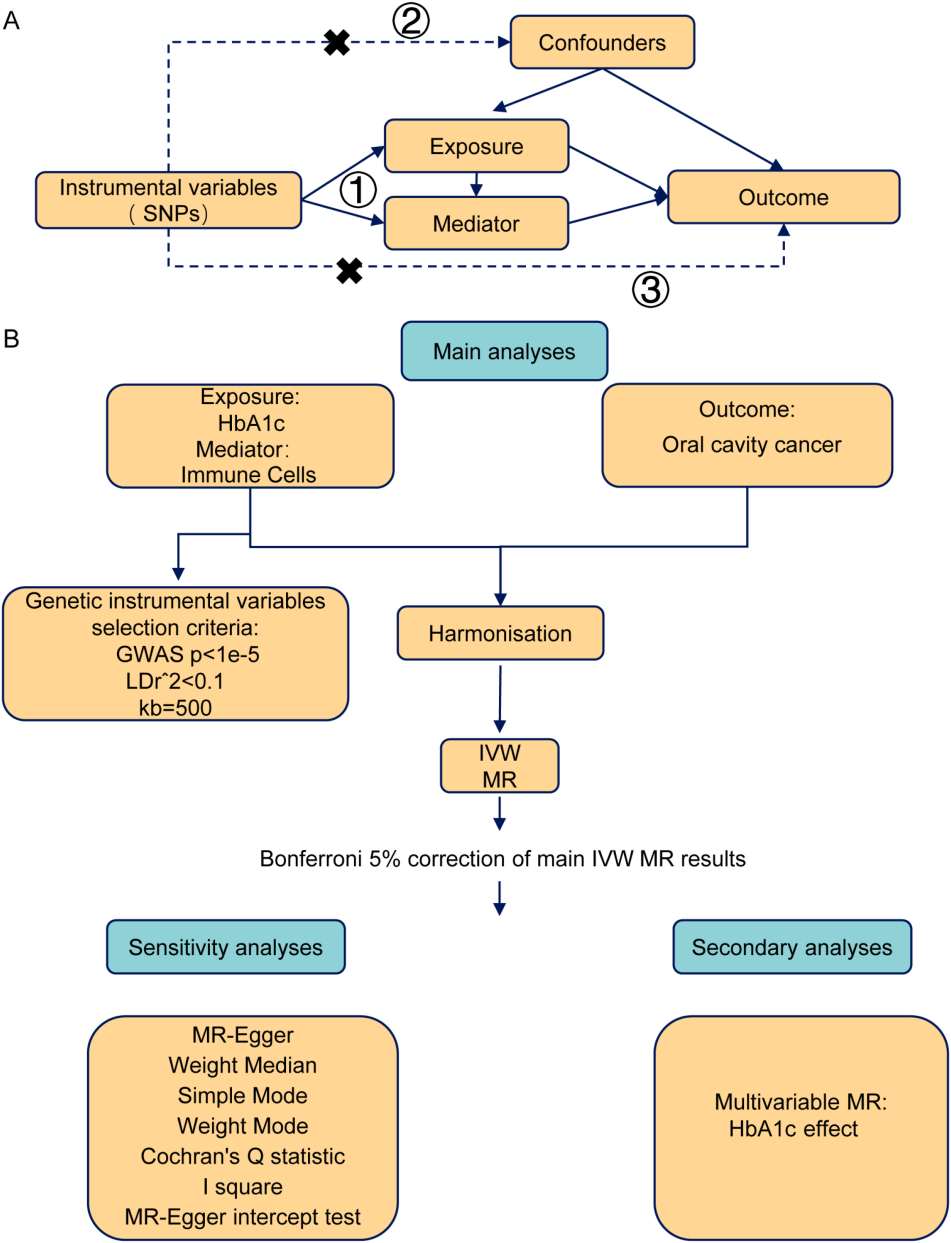
Flow chart of the causal inference study of immune cell–mediated effects of HbA1c on oral cavity cancer. **(A)** Mendelian randomization study design: 1) SNP selection and instrumental variable validation (F statistics); 2) two-sample MR analysis of HbA1c and oral cavity cancer (IVW, MR-Egger, weighted median, simple and weighted mode); 3) reverse MR analysis. **(B)** Immune cell–mediated analysis and experimental validation using multivariable MR and in vitro assays with AGEs.

A two-sample Mendelian randomization design was applied to estimate the causal effect of HbA1c levels on the risk of oral cavity cancer. We also explored how immune cells mediate the pathogenesis of oral cancer linked to HbA1c. We adhered to the STROBE-MR guidelines to ensure comprehensive reporting of this Mendelian randomization study (15). The study design is schematically illustrated in **Figure 1**.

### 2.1 Data sources

A large scale multi-phenotypic GWAS meta-analysis, which comprised 46368 individuals of study by Soranzo N et al (16), yielded summary GWAS data for HbA1c. Summary statistics for oral cancer were obtained from a multi-phenotype meta-analysis by Gormley M et al (17). The GWAS data adopted in this study contained 372373 individuals, including 357 cases and 372016 controls. Immune cell GWAS data was derived from the GWAS analysis performed by Sun BB et al (18) on the relevant data of 7313 subjects of European ancestry. The list of immune cells was originated from immune cell-related research literature (19) and the GWAS analysis data of 731 immune cells were obtained (**Supplementary Table S1**).

### 2.2 Instrumental variable selection

A valid instrumental variable (IV) is required to meet three fundamental criteria. Firstly, the chosen IVs should exhibit a robust association with the exposure variable. Secondly, these IVs must remain uncorrelated with any potential confounding variables that may affect either the exposure or the outcome. Lastly, the IV should exclusively influence the outcome through its impact on the exposure.

In current study, the IVs were screened according to the following criteria: SNPS with P< 1×10–5 for Single Nucleotide polymorphisms (SNPS) in GWAS were selected as the primary screening criteria, and SNPS in linkage disequilibrium (SNPS with r< 2 0.1 and physical distance > 500 kb between every two genes) were excluded. Subsequently, instrumental variables were derived from the GWAS of the outcome data based on the chosen SNPs. Concurrently, F-statistics were computed to evaluate the potential for weak instrumental variable bias. An F-value of less than 10 signifies that the genetic variation employed functions as a weak instrumental variable, potentially introducing bias into the findings; therefore, it should be excluded to prevent distortion of the results. The calculation formula for the F-statistic is presented below:


$$\text{F}=\frac{\text{N}-\text{K}-1}{\text{K}}*\frac{{\text{R}}^{2}}{1-{\text{R}}^{2}}$$

In this formula, N means the sample size, K indicates the number of instrumental variables used, and R2 reflects the extent to which the instrumental variables explain the exposure. R2 = 2 × (1-MAF) ×MAF×2β. In the formula, MAF indicates the minimum allele frequency and β means the allele effect size. SNP harmonization between exposure and outcome datasets was performed using the TwoSampleMR framework. Palindromic variants with ambiguous strand orientation were excluded when allele alignment could not be reliably resolved. Linkage disequilibrium clumping was conducted independently for each exposure dataset using a European reference panel to ensure SNP independence. A significance threshold of *P* < 1 × 10^−5^ was applied to balance instrument strength and trait coverage, which is consistent with prior Mendelian randomization studies of immune-related traits.

### 2.3 Cell viability assay (CCK-8)

Cell viability of Cal-27 and SAS cells treated with 50, 100, or 200 μg/ml BSA or AGEs (Biogradetech, USA) was measured with a CCK-8 kit (Dojindo, Japan). AGEs were used as experimental proxies for chronic glycemic exposure, as they represent stable downstream products of sustained hyperglycemia and are closely linked to HbA1c levels. This approach allows functional assessment of glycation-related metabolic stress on immune cell behavior.

After 12–60 h of incubation, 10 μL of CCK-8 solution was added per well, followed by a 2 h incubation at 37°C. Absorbance at 450 nm was then recorded on a BioTek microplate reader. All conditions included ≥5 replicates and were independently repeated in triplicate.

### 2.4 Flow cytometry

CD8^+^ T cells were isolated from peripheral blood mononuclear cells (PBMCs) using flow cytometric sorting. After the CD8^+^ T cells were sorted, they were activated under the condition of T cell receptor stimulation (using anti-CD3/CD28 antibodies), and then were co-treated with 100 μg/mL BSA or AGEs for 48 hours. Prior to harvesting, Brefeldin A was added to the culture for the final several hours to inhibit protein transport. Cells were then harvested, surface-stained for CD8, fixed, permeabilized, and intracellularly stained for IFN-γ and GZMB with fluorochrome-conjugated antibodies (**Supplementary Figure S1**). Data acquisition was performed on a flow cytometer, and analysis was conducted using FlowJo software.

### 2.5 Cytotoxicity assay (LDH release)

Cytotoxicity of effector cells against OSCC cells was quantified using an LDH assay kit (Thermo Fisher Scientific). Cal-27 and SAS cells were seeded in 48-well plates and pre-treated with 100 μg/ml BSA or AGE-BSA for 48 hours prior to the co-culture. Subsequently, effector cells activated T cells) were added to the target cells at E:T ratios of 1:1, 5:1, 10:1, and 20:1. After co-culturing for 48 hours, the supernatant was collected, and the LDH activity was measured. The percentage of specific cell lysis (% Lysis) was calculated using the formula provided by the kit.

### 2.6 Measurement of intracellular reactive oxygen species

CD8^+^ T cells treated with BSA or AGEs (100 μg/ml) in RPMI 1640 medium for 48 h were incubated with the ROS-sensitive fluorescent probe DCFH-DA for 30 min at 37 °C in the dark. After washing and resuspension in PBS, cells were immediately subjected to flow cytometric analysis (BD FACSCalibur), and the percentage of ROS-positive cells was quantified using FlowJo software.

### 2.7 Statistical analysis

Statistical analyses were conducted in R (v4.2.2) using the TwoSampleMR package for Mendelian randomization and two-way ANOVA for *in vitro* assays (CCK-8, LDH). MR sensitivity analyses included Cochran’s Q test, leave-one-out analysis, and MR-Egger intercept test. Results are expressed as odds ratios (ORs) with 95% confidence intervals (CIs). Significance was defined as *P* < 5×10–^6^ for GWAS SNPs and Bonferroni-corrected *P<* 0.05 for other tests; all *P*-values were two-sided.

Multiple two-sample MR methods were applied, including IVW, MR-Egger, weighted median, simple mode, and weighted mode, with IVW serving as the primary analysis due to its robustness (20), which stems from its fitting of a regression model without an intercept term and its use of the inverse outcome variance as weights (21). The IVW method served as the primary analysis in the absence of pleiotropy, supplemented by other approaches. The choice between random- or fixed-effects IVW models was determined by the presence of heterogeneity. In instances of pleiotropy, the MR-Egger regression method ensured the integrity of the MR approach, particularly in studies involving multiple genetic variants as instrumental variables. Additionally, reverse causality was investigated to assess potential causal influences on exposure using the same methodological framework.

Sensitivity analyses assessed heterogeneity (Cochran’s Q and I² statistic), directional pleiotropy (MR-Egger intercept test, where P > 0.05 indicates none), and the influence of individual SNPs (leave-one-out analysis). A notable change in the causal estimate upon excluding a single SNP suggested that the result was influenced by that variant.

Multivariable MR (MVMR) was conducted to estimate the direct causal effect of each exposure on the outcome. This method extends univariable MR by using genetic instruments associated with multiple interrelated exposures to assess their independent effects. The direct effects of HbA1c and immune cells on oral cancer were derived from MVMR, whereas univariable MR was applied to evaluate the indirect effect of HbA1c mediated through immune cells.

## 3 Results

### 3.1 Selection of instrumental variables

Following the IV selection criteria, linkage disequilibrium (LD) clumping was applied to ensure SNP independence. Following this, a data matching process was performed utilizing GWAS data pertinent to oral cancer, resulting in the inclusion of 2,529,804 SNPs that were linked to HbA1c levels as instrumental variables. Ultimately, 7,723,107 SNPs were identified as the definitive instrumental variables relevant to oral cavity cancer. The instrumental variables (genetic variants) used in this study are listed in **Table 1**, which includes only those yielding statistically significant MR results (*P* < 0.05). The F-statistics for HbA1c-associated SNPs ranged from 19.53 to 268.52 (median = 26.31), all well above the conventional threshold of 10, indicating minimal weak instrument bias.

**Table 1:** The sources of GWAS data.

Exposure	GWAS ID	Consortium	Ethnicity	Sample sizes	Number of SNPs	Sex	Year
HbA1C	ieu-b-104	MAGIC	European	46368	2529804	Males and Females	2010
Oral cavity cancer	ieu-b-4961	UK Biobank	European	372373	7723107	Males and Females	2021

### 3.2 Causal effect of HbA1c on oral cavity cancer

**Figure 2:**
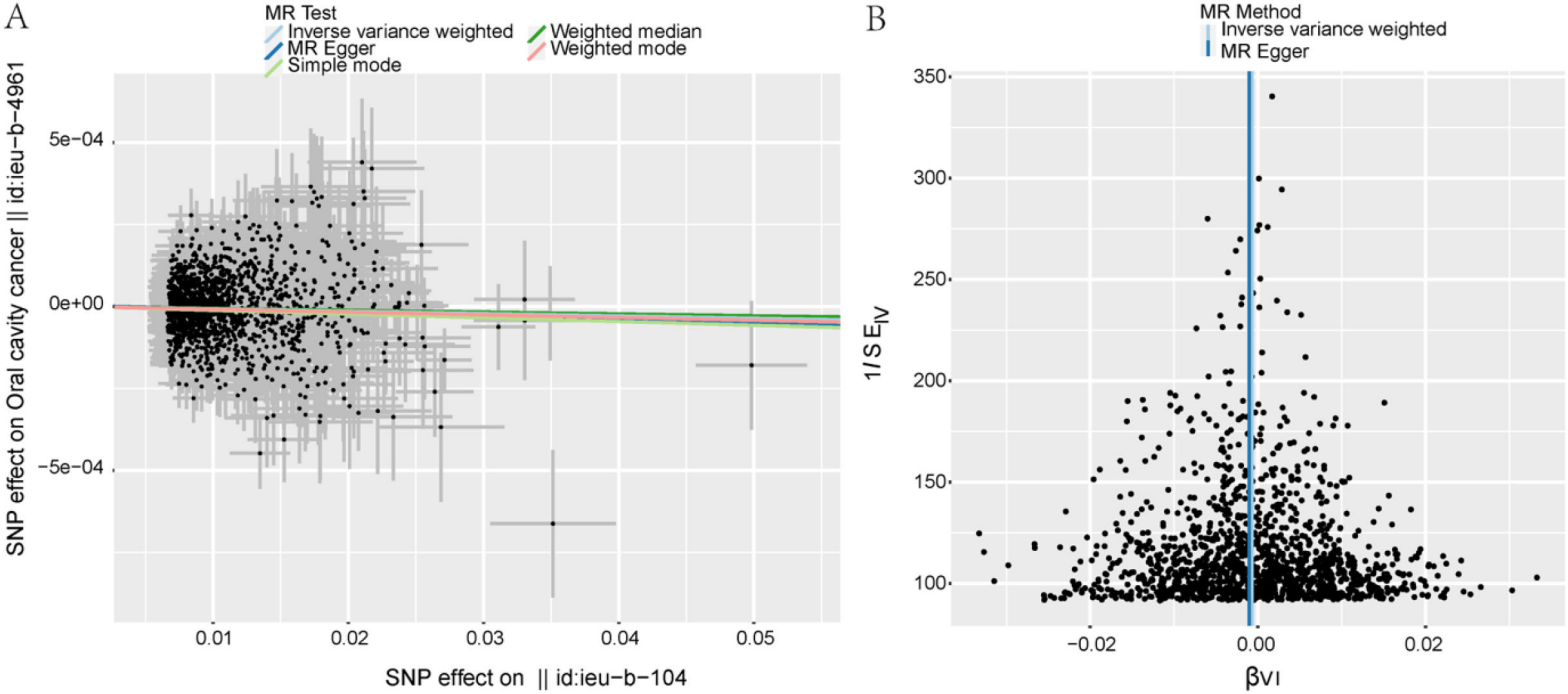
Mendelian random causal effect estimation of HbA1c on the risk of oral cancer. **(A)** Scatter plots, the slopes of the lines indicate the magnitude of the causal relationship predicted by the different models; **(B)** the funnel plot, the causal effect estimates for the Inverse Variance Weighted and MR-Egger models are represented by straight lines in the plots.

IVW analysis revealed a significant inverse association between genetically predicted HbA1c and oral cancer risk (OR = 0.999, 95% CI: 0.999–1.000, P = 0.002). Scatter and funnel plots (**Figures 2A, B**) showed generally aligned slope directions and symmetrical effect estimates, indicating no substantial bias. Consistency across MR models was observed, with the IVW intercept near zero.

### 3.3 Sensitivity analyses

Sensitivity analyses indicated low heterogeneity among HbA1c-associated SNPs (Cochran’s Q = 1497.83, P = 0.018; I² = 7.47%) and no evidence of directional pleiotropy (MR-Egger intercept P = 0.620). The leave-one-out analysis identified no influential SNPs, confirming the stability of the MR results (**Supplementary Table S2**).

### 3.4 Reverse MR analysis

**Figure 3:**
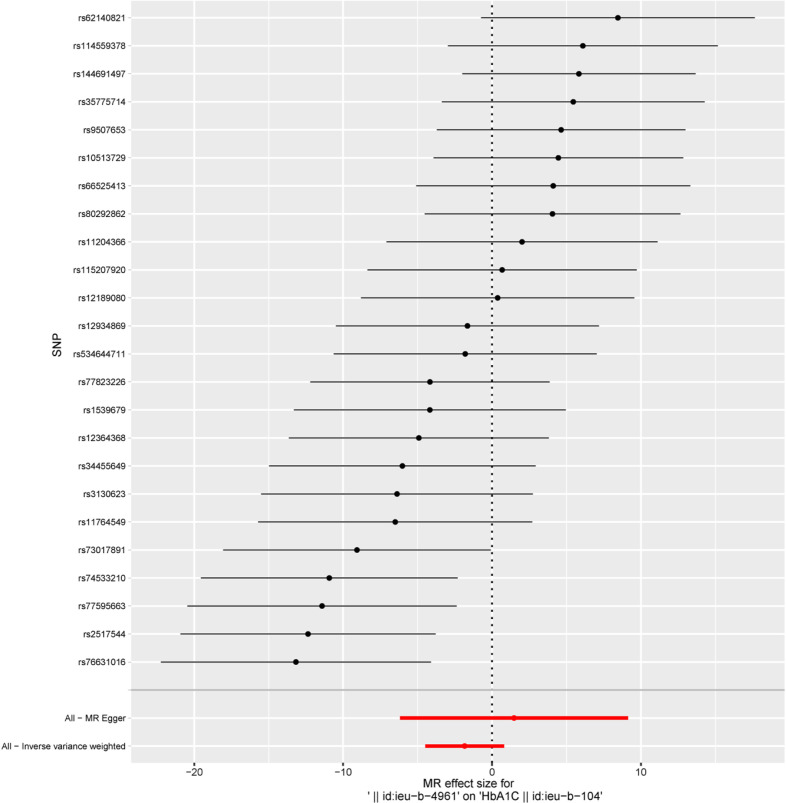
The forest plot showing reverse MR Analysis indicates that oral cancer has no causal effect on Hba1c.

Reverse MR analysis indicated no causal effect of oral cancer on HbA1c levels (IVW OR = 0.159, 95% CI: 0.011–2.242, P = 0.173). Consistency across supplementary methods (MR-Egger, weighted median, simple mode, weighted mode) further ruled out reverse causality, as visualized in the forest plot (**Figure 3**). Reverse MR analysis revealed no bidirectional association, suggesting HbA1c as an independent risk factor.

### 3.5 Multivariate MR analysis and mediating effect estimation

Multivariable Mendelian randomization was applied to estimate the direct effects of HbA1c and immune cell traits on oral cavity cancer. It should be noted that this approach evaluates the independent contributions of correlated exposures and does not, by itself, provide definitive evidence of mediation. The observed associations therefore suggest potential immune-related pathways through which HbA1c may influence oral cavity cancer risk, rather than confirming a fully mediated causal mechanism (**Table 2**). The findings indicated eight distinct groups of HbA1c with significant causal effects on immune cells, all confirmed by the Steiger directivity test as TRUE, thus ruling out reverse causality. Additionally, the leave-one-out analysis found no potential outlier SNPs between HbA1c and oral cavity cancer, confirming the robustness of the results (**Table 3**). Notably, model 2, model 6, and model 7 exhibited statistically significant relationships with oral cancer (P<0.05), suggesting that HbA1c might influence outcomes through these specific pathways which potentially mediate its effects. No significant differences in outcomes were observed among models 1, 3, 4, 5 and 8. However, it is worth mentioning that these models demonstrated odds ratio values close to unity, further experimental verification is required.

**Table 2:** Inverse variance weighted random-effects model analysis of HbA1C and immune cells.

Exposure	Outcome	β	Standard error	P value	OR (95% CI)
HbA1C	Effector Memory CD8+ T cell Absolute Count || id:ebi-a-GCST90001554	-0.238627619	0.083364792	0.004203785	0.787708155080822(0.66896561903889,0.927527693384733)
HbA1C	Terminally Differentiated CD8+ T cell Absolute Count || id:ebi-a-GCST90001557	-0.248181971	0.083248518	0.002871044	0.780217952996568(0.662755545182624,0.918498620800546)
HbA1C	Lymphocyte Absolute Count || id:ebi-a-GCST90001601	-0.265210826	0.081800102	0.001186182	0.767044219772717(0.653417481507453,0.900430202340743)
HbA1C	B cell %lymphocyte || id:ebi-a-GCST90001644	-0.211694934	0.080953321	0.008922101	0.80921152298974(0.690483333166786,0.948354952951779)
HbA1C	CD28- CD8+ T cell %T cell || id:ebi-a-GCST90001685	-0.171090033	0.078437377	0.02916634	0.842745694723519(0.72265215010084,0.982796918096654)
HbA1C	CD28- CD8+ T cell Absolute Count || id:ebi-a-GCST90001687	-0.227304558	0.082779109	0.006034204	0.796678110355553(0.677360526633547,0.937013579273813)
HbA1C	CD20 on unswitched memory B cell || id:ebi-a-GCST90001760	0.260320293	0.082379424	0.001577605	1.29734555051994(1.10390801072246,1.52467910469493)
HbA1C	CD45 on granulocyte || id:ebi-a-GCST90001913	-0.213705354	0.085146741	0.012078323	0.807586301907819(0.683456022957113,0.954261303027646)

**Table 3:** The results of multivariable Mendelian randomization analysis on the impact of HbA1c trait and oral cavity cancer.

Model	Exposure	Outcome	OR (95%CI)	P value
Model 1	CD45 on granulocyte || id:ebi-a-GCST90001913	Oral cavity cancer || id:ieu-b-4961	1.000 (1.000, 1.000)	0.277781832
	HbA1C || id:ieu-b-104	Oral cavity cancer || id:ieu-b-4961	0.999 (0.999, 1.000)	0.011125404
Model 2	Effector Memory CD8+ T cell Absolute Count || id:ebi-a-GCST90001554	Oral cavity cancer || id:ieu-b-4961	1.000 (1.000, 1.000)	0.024281525
	HbA1C || id:ieu-b-104	Oral cavity cancer || id:ieu-b-4961	0.999 (0.999, 1.000)	0.013254165
Model 3	Terminally Differentiated CD8+ T cell Absolute Count || id:ebi-a-GCST90001557	Oral cavity cancer || id:ieu-b-4961	1.000 (1.000, 1.000)	0.060360158
	HbA1C || id:ieu-b-104	Oral cavity cancer || id:ieu-b-4961	0.999 (0.999, 1.000)	0.015219133
Model 4	Lymphocyte Absolute Count || id:ebi-a-GCST90001601	Oral cavity cancer || id:ieu-b-4961	1.000 (1.000, 1.000)	0.870957849
	HbA1C || id:ieu-b-104	Oral cavity cancer || id:ieu-b-4961	0.999 (0.999, 1.000)	0.009049505
Model 5	B cell %lymphocyte || id:ebi-a-GCST90001644	Oral cavity cancer || id:ieu-b-4961	1.000 (1.000, 1.000)	0.143160256
	HbA1C || id:ieu-b-104	Oral cavity cancer || id:ieu-b-4961	0.999 (0.999, 1.000)	0.006388427
Model 6	CD28- CD8+ T cell %T cell || id:ebi-a-GCST90001685	Oral cavity cancer || id:ieu-b-4961	1.000 (1.000, 1.000)	0.007096049
	HbA1C || id:ieu-b-104	Oral cavity cancer || id:ieu-b-4961	0.999 (0.999, 1.000)	0.014151919
Model 7	CD28- CD8+ T cell Absolute Count || id:ebi-a-GCST90001687	Oral cavity cancer || id:ieu-b-4961	1.000 (1.000, 1.000)	0.004692039
	HbA1C || id:ieu-b-104	Oral cavity cancer || id:ieu-b-4961	0.999 (0.999, 1.000)	0.021691411
Model 8	CD20 on unswitched memory B cell || id:ebi-a-GCST90001760	Oral cavity cancer || id:ieu-b-4961	1.000 (1.000, 1.000)	0.74505017
	HbA1C || id:ieu-b-104	Oral cavity cancer || id:ieu-b-4961	0.999 (0.999, 1.000)	0.009381104

### 3.6 Effects of BSA and AGEs on cell viability in OSCC cells

**Figure 4:**
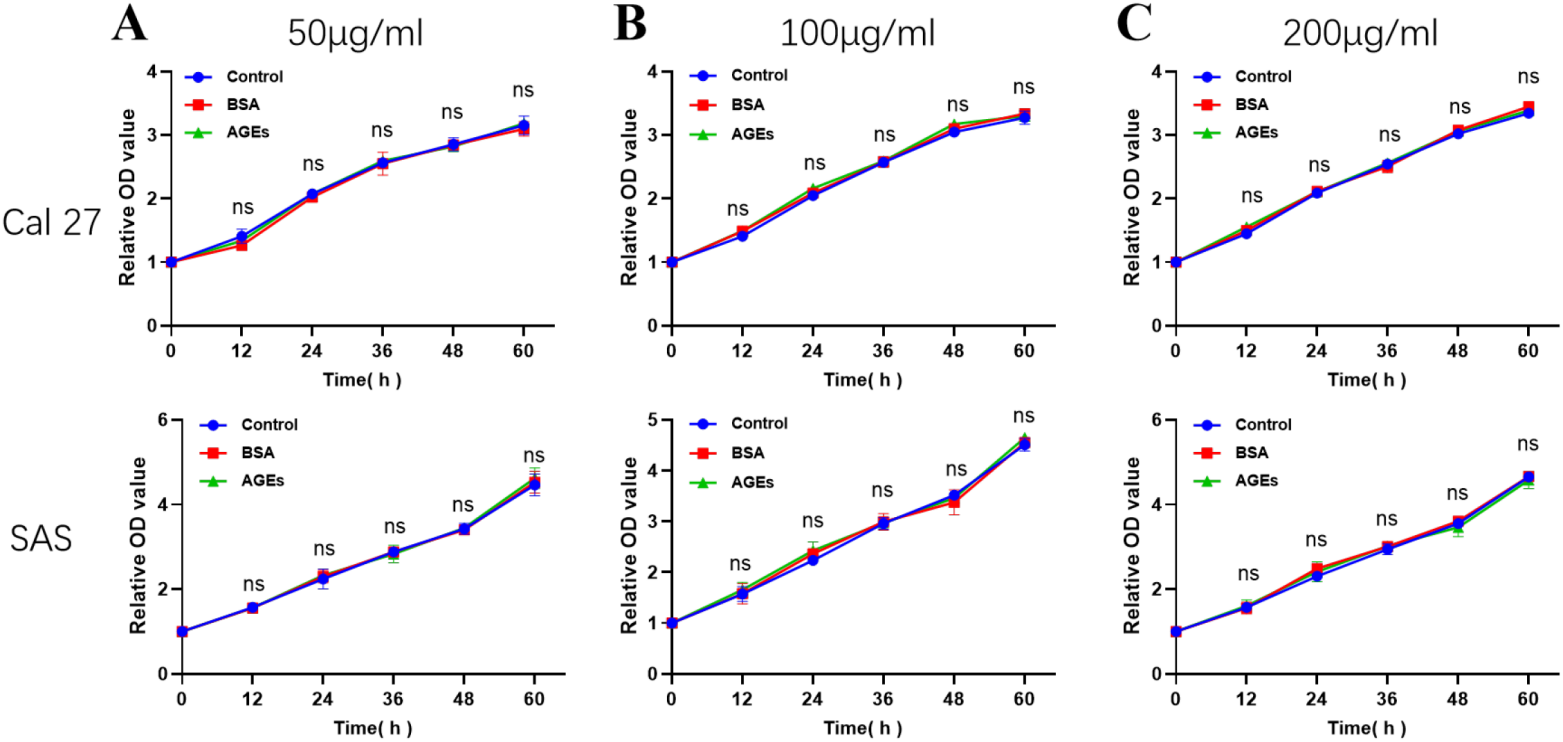
Effects of BSA and AGEs on the viability of Cal 27 and SAS cells. Cells were treated with **(A)** 50 μg/ml, **(B)** 100 μg/ml, or **(C)** 200 μg/ml of BSA or AGEs for 12, 24, 36, 48, and 60 hours. Cell viability was assessed by the CCK-8 assay and is expressed as a percentage relative to the control group. Data are presented as mean ± SD from three independent experiments.

To investigate the impact of BSA and AGEs on the viability of OSCC cells, Cal 27 and SAS cells were treated with varying concentrations (50, 100, and 200 μg/ml) of BSA or AGEs for different durations (12, 24, 36, 48, and 60 hours). The CCK-8 assay was employed to assess cell viability. As shown in **Figure 4**, different concentrations of BSA or AGEs had no significant effects on cell viability during the treatment.

### 3.7 AGEs suppress the expression of effector molecules in CD8+ T cells

**Figure 5:**
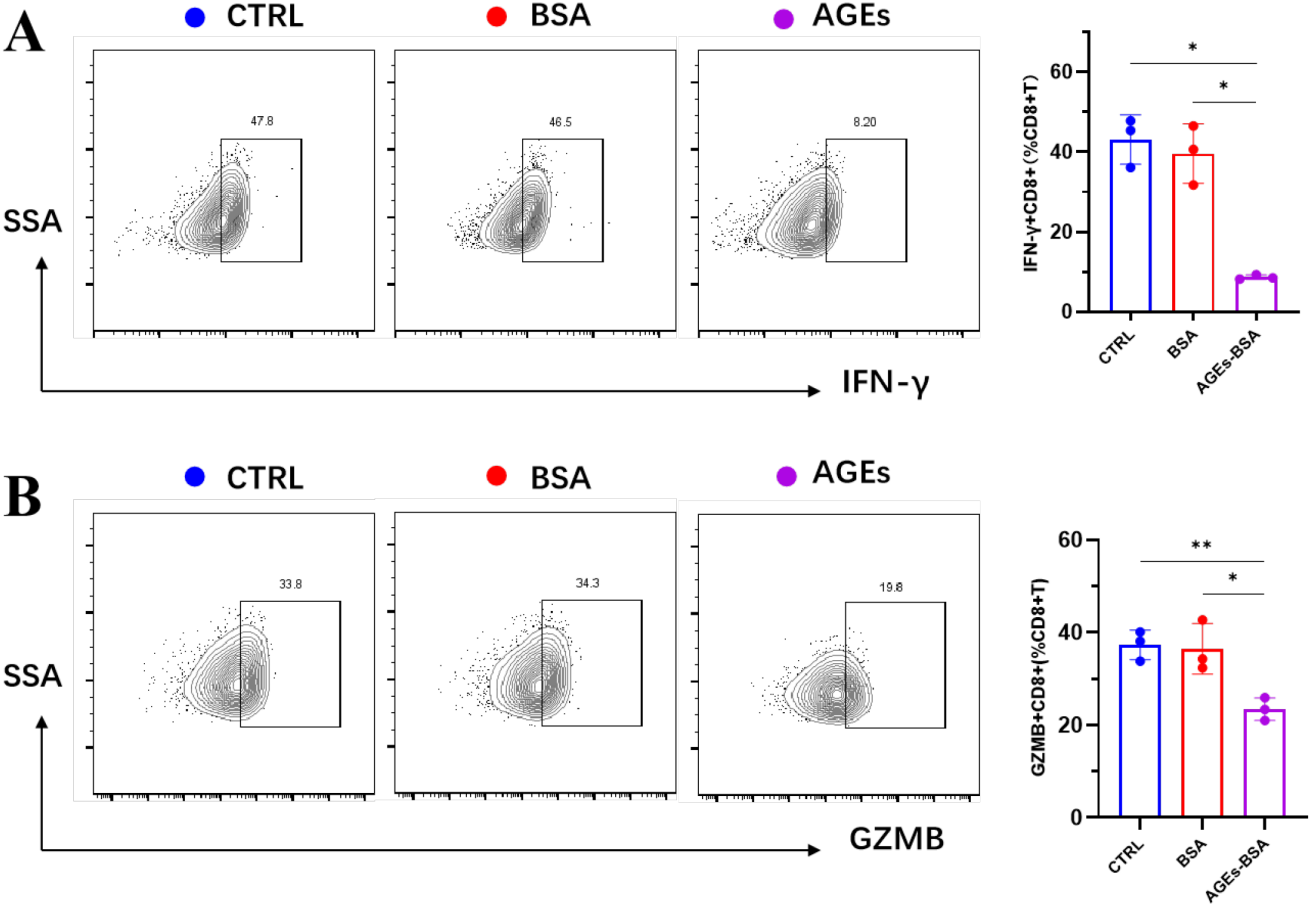
AGEs inhibit IFN-γ and GZMB expression in CD8+ T cells. CD8+ T cells were treated with BSA or AGEs. The intracellular expression of **(A)** IFN-γ and **(B)** GZMB was analyzed by flow cytometry. Representative flow cytometry plots (left) and quantitative summary data (right) are shown. Data are presented as mean ± SD. ^*^p < 0.05, ^**^p < 0.01 vs. Control and BSA groups.

Based on the results of the aforementioned multivariate Mendelian randomization analysis, CD8+ T cells may serve as a key mediator in the association between HbA1c and oral cancer. Consequently, we further examined the impact of AGEs on CD8+ T cell function. We quantified the intracellular expression of IFN-γ and GZMB by flow cytometry. As shown in **Figures 5A, B**, AGEs treatment significantly reduced the proportions of both IFN-γ-positive and GZMB-producing CD8^+^ T cells compared to the untreated control and BSA-treated groups (all *p* < 0.05).

### 3.8 AGEs impair effector cell-mediated cytotoxicity against OSCC cells

To determine whether AGEs affect the susceptibility of oral cancer cells to effector cell-mediated killing, we performed a lactate dehydrogenase (LDH) release assay. Cal 27 and SAS cells, pre-treated with 100 μg/ml BSA or AGE-BSA, were co-cultured with effector cells at varying effector-to-target (E:T) ratios.

**Figure 6:**
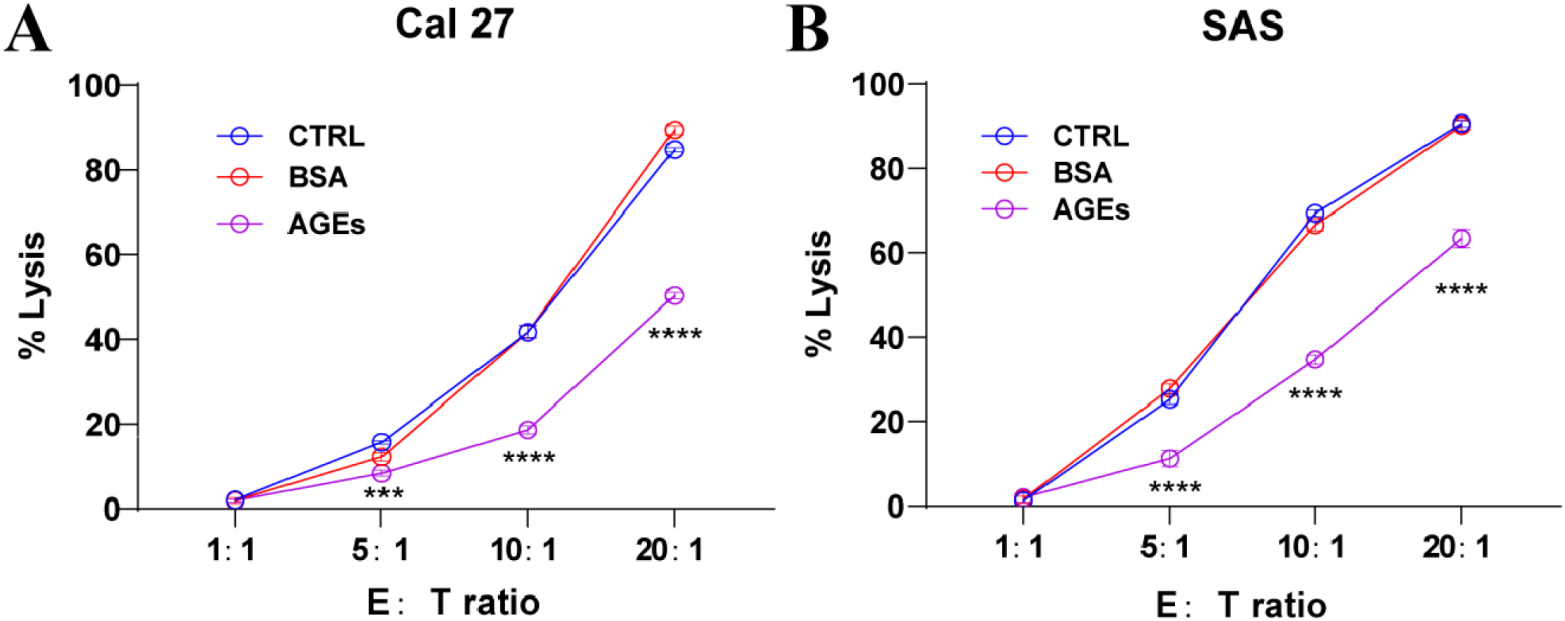
AGE-BSA treatment reduces the susceptibility of OSCC cells to effector cell-mediated cytotoxicity. Cal 27 **(A)** and SAS **(B)** cells were pre-treated with 100 μg/ml BSA or AGE-BSA for 24 hours before being co-cultured with effector cells at the indicated E:T ratios. Specific cell lysis (% Lysis) was determined by an LDH release assay. Data are presented as mean ± SD from at least three independent experiments. ***p < 0.001, ^****^p < 0.0001 indicates a statistically significant difference compared to both the Control and BSA groups at the same E:T ratio.

As shown in **Figure 6A**, in Cal 27 cells, the specific lysis (% Lysis) was significantly reduced in the AGE-BSA treated group compared to the untreated control and BSA-treated groups when the E:T ratio reached 5:1, 10:1 and 20:1 (*p* < 0.0001).

A similar effect was observed in SAS cells (**Figure 6B**). At the highest E:T ratio of 5:1, 10:1 and 20:1, pre-treatment of SAS cells with AGE-BSA resulted in a marked decrease in % Lysis compared to both control groups (*p* < 0.0001).

### 3.9 AGEs induce oxidative stress in CD8+ T cells

**Figure 7:**
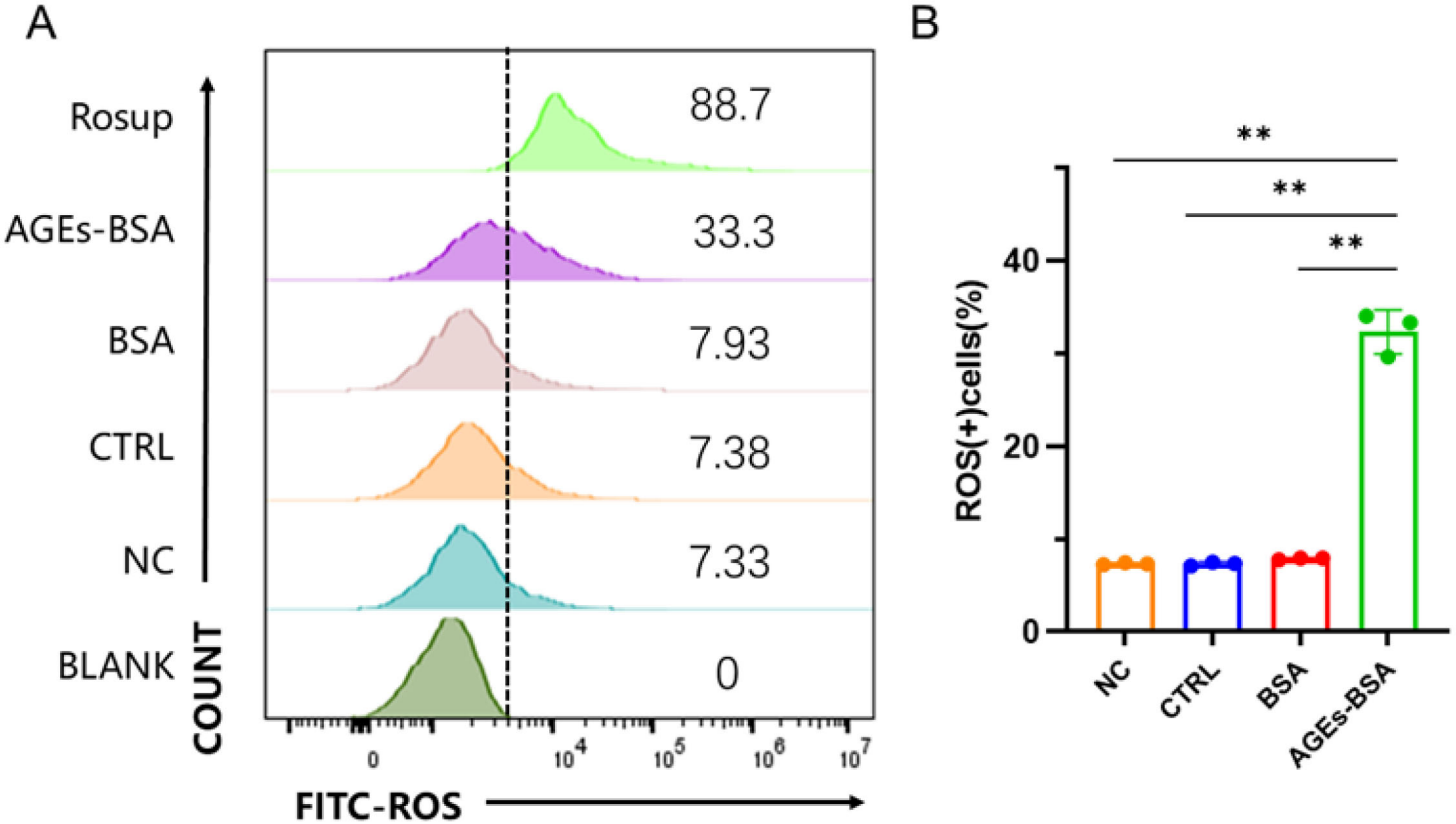
AGEs induce oxidative stress in CD8+ T cells. **(A)** Representative flow cytometry plots showing intracellular ROS levels in CD8+ T cells treated with BSA or AGEs (100 μg/ml) for 48 hours. **(B)** Quantitative summary of the percentage of ROS-positive cells. Data are presented as mean ± SD from three independent experiments. ^**^p < 0.01 compared to Control and BSA groups.

We further investigated whether AGEs influence intracellular reactive oxygen species (ROS) levels in CD8+ T cells. As shown in **Figures 7A, B**, flow cytometric analysis revealed a significant increase in ROS-positive cells following AGEs treatment compared to the BSA and untreated control groups (*p* < 0.01). These results indicate that AGEs enhance oxidative stress in CD8+ T cells, which may contribute to their functional impairment.

## 4 Discussion

Malignant tumors and diabetes constitute major global health challenges, with cancer ranking as a leading cause of mortality worldwide (22). Epidemiological evidence supports a link between diabetes and a higher risk of oral cancer (23). Nonetheless, these studies are constrained by limitations such as limited sample sizes and the influence of confounding variables, including tobacco use, alcohol consumption, betel nut intake, and anti-diabetic medications. Metabolomic signatures associated with diabetes are considered to have potential predictive value for cancer outcomes (24). HbA1c is the gold standard for assessing long-term (5–10 year) risks of micro- and macrovascular complications in diabetes (25). Elevated HbA1c levels have been linked to an increased risk of multiple cancers (26) and are also an independent risk factor for diabetes (27). Given its role as a marker of chronic hyperglycemia, it would be valuable to explore the prevention and treatment strategies for oral cancer, which we investigate here.

This study employed a bidirectional two-sample MR design to investigate the causal link between HbA1c and oral cancer. To our knowledge, this represents the first application of MR to elucidate this relationship, addressing a significant gap in the literature. By using a large public dataset, our analysis offers new insights into the relationship between these factors and oral cancer. Our findings suggest that elevated levels of HbA1c might influence the risk of oral cancer through immune cell-mediated mechanisms. To our knowledge, this is also the first study to examine the mediating role of immune cells in linking HbA1c with oral cancer. Our findings provide mechanistic insight into oral cancer pathogenesis and position HbA1c as a candidate biomarker for early detection, highlighting the metabolic-immune interface in oncogenesis.

Although the diabetes-cancer link has long been recognized, establishing causality remains challenging for observational studies due to residual confounding and difficulty in delineating temporal sequence, limiting insight into glucose’s specific role in oral cancer. In this study, genetic variation is employed as a means to simulate randomized trials, thereby mitigating confounding factors and offering more reliable causal inferences to compensate for the constraints associated with observational studies. The sample size utilized in the MR Study was derived from a comprehensive multi-phenotypic GWAS meta-analysis, ensuring an adequate representation (28). In Mendelian randomization analyses, the estimated effect size reflects the impact per genetically predicted unit increase in HbA1c, as defined in the original GWAS. Although the resulting odds ratio appears numerically close to unity, such small effect sizes are common in genetically scaled exposures and may represent cumulative lifetime biological effects rather than acute or clinically large changes in glycemic status.

A causal relationship between HbA1c levels and oral cancer risk was identified through two-sample MR, indicating HbA1c’s promise as a biomarker for risk assessment. Previous studies have demonstrated that HbA1c may influence cancer development through multiple mechanisms. HbA1c, a product of non-enzymatic hemoglobin glycation, integrates average glycemic exposure over 2–3 months and is positively associated with insulin resistance (29). Insulin resistance-induced abnormal glucose metabolism may promote cellular proliferation and inhibit apoptosis, thereby potentially increasing the risk of tumorigenesis (30). In addition, the elevation of HbA1c levels is intricately linked to increased oxidative stress (31). Elevated HbA1c not only contributes to heightened oxidative stress in diabetic patients but also directly induces DNA damage, promotes cell proliferation, inhibits apoptosis, and alters the tumor microenvironment (32, 33). Moreover, increased HbA1c levels are associated with a rise in reactive oxygen species (ROS) generation, which can trigger cellular damage and mutations, thereby facilitating cancer development (32). Elevated HbA1c is associated with a chronic inflammatory phenotype, marked by increased cytokines like TNF-α and IL-6, which further aggravates insulin resistance and dysglycemia (34). In diabetic patients, elevated HbA1c levels are often accompanied by upregulated expression of apoptosis-related markers, including cleaved caspase-3 and caspase-7 (35). Additionally, increased HbA1c levels are associated with the activation of other cell death pathways, such as autophagy and necrosis, which may play significant roles in the pathogenesis of diabetes-associated tumors (36). The observed association in this study aligns with previous studies indicating that metabolic dysregulation could impact cancer progression, thereby highlighting the possibility for HbA1c to play a role in the intricate interplay between diabetes and cancer.

Our *in vitro* experiments functionally substantiate the MR findings that glycation products may influence oral cancer biology through immune modulation rather than direct tumoricidal effects. We demonstrated that neither AGEs nor BSA significantly affected OSCC cell viability, suggesting that the protective effect of elevated HbA1c is not mediated through direct modulation of tumor cell proliferation. More importantly, we identified a novel immunomodulatory mechanism: AGEs directly impaired CD8^+^ T cell function, as evidenced by significantly reduced production of the key effector molecules IFN-γ and Granzyme B.

Rather than representing a contradiction, the Mendelian randomization and *in vitro* findings likely reflect different temporal windows of glycemic influence. Genetically predicted HbA1c captures long-term systemic metabolic regulation, whereas advanced glycation end products represent downstream consequences of sustained hyperglycemia that may act at later stages. In this context, immune compensation mechanisms and dynamic changes in immune function over time may differentially influence cancer susceptibility, providing a plausible framework to reconcile the genetic and experimental observations. Furthermore, AGEs treatment markedly elevated intracellular ROS levels in CD8^+^ T cells (**Figure 7**), indicating that oxidative stress may be a key mechanism underlying their functional impairment. The observed T cell exhaustion subsequently led to a profound reduction in the cytotoxicity of effector cells against OSCC cells. These results mechanistically bridge the gap between elevated HbA1c levels and impaired anti-tumor immunity, suggesting that AGEs accumulation under hyperglycemic conditions could foster an immunosuppressive microenvironment by inducing CD8^+^ T cell dysfunction through oxidative stress, thereby potentially promoting oral carcinogenesis despite the observed protective association. This apparent paradox may reflect the complex temporal dynamics of immune responses during carcinogenesis, where early immune suppression might paradoxically reduce chronic inflammation-driven carcinogenesis in specific contexts. These findings indicate that immune cell modulation may contribute to the relationship between HbA1c and oral cavity cancer in a context-dependent manner, rather than serving as a single or definitive mediating mechanism.

Reverse MR analysis revealed no bidirectional causality, confirming that elevated HbA1c is an independent causal risk factor for oral cancer. HbA1c is a potential risk factor for oral cancer independent of diabetes, challenging the traditional understanding of the metabolic-cancer relationship and highlighting the importance of diabetes management in cancer prevention strategies.

Our analysis further indicated that elevated HbA1c influences multiple immune cell subsets (e.g., activated B and T cells), with eight types showing significant associations (P< 0.05), suggesting immunomodulation as a potential mechanism. This is notable given the dual role of immunity in cancer control and the recognized immune dysfunction in diabetes due to hyperglycemia and insulin deficiency (37). Defective innate immune responses (including neutrophil and macrophage dysfunction) as well as impaired adaptive immunity (including T cell dysfunction) are believed to contribute to compromised overall immune system function among individuals with diabetes (38). These *in vitro* experiments provide mechanistic support for immune modulation associated with glycation-related metabolic stress; however, they do not directly replicate the complexity of the *in vivo* tumor microenvironment and should be interpreted accordingly.

Recent evidence indicates that hyperglycemia in diabetes is associated with elevated pro-inflammatory cytokines (e.g., IL-6, TNF-α) and reduced levels of key immune cells such as CD4+ and CD8+ T cells (39). This relationship implies that blood glucose levels may indirectly influence tumor initiation and progression by modulating immune cell function. T-cell functionality is compromised in diabetic patients, characterized by diminished cellular proliferation and reduced cytokine secretion. This phenomenon is closely tied to oxidative stress and metabolic disturbances caused by hyperglycemia. Specifically, elevated blood glucose levels induce mitochondrial dysfunction in T cells and enhance fatty acid synthesis, leading to oxidative stress and lipid peroxidation, ultimately resulting in the degradation of the signal transduction protein *STAT4*, thereby inhibiting T cell differentiation and function (40). A study utilizing a diabetic mouse model to elucidate the distinct immune landscapes within euglycemic and hyperglycemic tumor microenvironments demonstrated that elevated glucose levels upregulate PD-L1 expression via Ras-signaling-mediated PTRH1 downregulation, consequently suppressing T cell cytotoxic function in the tumor environment (41). Moreover, fluctuations in blood glucose levels directly impact macrophage polarization, which in turn influences inflammatory responses and immune regulation (42). Immune cells play a dual role in tumorigenesis, capable of both inhibiting tumor growth and promoting tumor progression (43). Fluctuations in blood glucose levels can influence the functionality of immune cells, potentially leading to impaired activity of these cells under hyperglycemic conditions (44). Therefore, it is crucial to comprehend the intricate interplay between blood glucose levels and the immune system.

The tumor microenvironment (TME) is composed of diverse immune cells—including T, B, NK, and dendritic cells, macrophages, neutrophils, mast cells, and MDSCs—that collectively shape antitumor immunity. Among these, CD8^+^ T cells are key effectors that recognize tumor antigens and execute cytotoxicity through cytokine release (e.g., IFN-γ, TNF-α) (45). Research has demonstrated a positive correlation between the infiltration of CD8+ T cells within tumors and patient survival rates, positioning CD8+ T cell infiltration as a prognostic marker for favorable outcomes across various cancers (46). Boosting CD8^+^ T cell activity is a central goal of cancer immunotherapy. CD4^+^ T cells critically regulate this process by modulating CD8^+^ T cell function, yet their frequent suppression in the tumor microenvironment enables immune evasion (47). Moreover, B cells contribute significantly to tumor immunity through antibody production. By secreting specific antibodies, they can recognize and neutralize tumor antigens, and enhance overall anti-tumor immunity via antigen presentation to activate T cells. However, alterations in the tumor microenvironment may impair B cell function and reduce their capacity for antibody production (48). Additionally, macrophages, dendritic cells (DCs), and natural killer (NK) cells regulate the tumor microenvironment through antigen presentation and cell-cell interactions (47). Understanding the mechanisms governing immune cell behavior in tumors holds substantial implications for therapeutic strategies. Our MVMR analysis results indicate that distinct immune cell types might exhibit varying degrees of involvement in the pathogenesis of oral cancer with respect to HbA1c levels. Specifically, significant differences (*P* < 0.05) observed in models 2, 6 and 7 suggest intricate interactions among immune cells that could influence oral cancer risk. It is worth noting that the effect size (OR value) of the above model is close to 1, suggesting a lack of clinical significance in the relationship between HbA1c and oral cancer through immune cells. The intricate causal pathways linking HbA1c and oral cancer might not be fully mediated by the specific immune cell models analyzed thus far. Nevertheless, our findings provide a basis for further investigation of the specific roles that these immune-cell populations play in disease progression and how they might guide personalized treatment strategies. Tailoring treatments to target specific immune pathways could enhance the efficacy of cancer interventions, particularly for patients with elevated HbA1c levels. Recognizing the contributions made by different immune cell subsets to oral cancer underscores the necessity for comprehensive studies simultaneously evaluating metabolic and immune parameters in cancer patients. Further functional studies are needed to elucidate the underlying biological mechanisms.

Despite the robustness of our findings, it is important to acknowledge the limitations of this study, particularly the absence of laboratory validation and clinical relevance. Although the sample size was substantial, encompassing 372373 individuals, potential selection bias and interstudy variability could impact the strength of our conclusions due to the diversity present in the dataset. The utilization of genetic tools to assess HbA1c levels introduces additional complexity as their applicability in different populations and backgrounds remains yet to be fully determined. Furthermore, a lack of stratification based on demographic and clinical characteristics might obscure subtle relationships that would provide greater insight into the association between HbA1c and oral cancer risk. Future studies should incorporate experimental designs and diverse cohorts to enhance both reliability and generalizability.

## 5 Limitations and conclusions

Several limitations should be acknowledged. Although the overall sample size of the GWAS datasets was large, the number of oral cavity cancer cases was relatively limited, which may influence the precision of effect estimates and the stability of multivariable analyses. This constraint may also limit the generalizability of the findings and underscores the need for future studies incorporating larger case numbers and complementary experimental validation.

In conclusion, this study provides compelling genetic and functional evidence that elevated HbA1c may confer a protective effect against oral cavity cancer through immune cell mediation. While our *in vitro* experiments showed that AGEs, the key derivatives of sustained hyperglycemia, did not significantly affect OSCC cell viability, we mechanistically demonstrate that they induce oxidative stress and potently impair CD8^+^ T cell effector functions and cytotoxicity. These findings elucidate a glycation-oxidative stress-immune axis linking glucose metabolism to oral carcinogenesis, offering valuable insights for future preventive and therapeutic strategies.

## Data Availability

The original contributions presented in the study are included in the article/**Supplementary Material**. Further inquiries can be directed to the corresponding author.
