# Supplementary figures and images for: Dissecting the causal pathway linking HbA1c to oral cavity cancer via immune cell modulation: a bidirectional mendelian randomization analysis
Source: Front Oncol. 2026 Feb 13;16:1768761. doi: 10.3389/fonc.2026.1768761 (PMC12945784; doi:10.3389/fonc.2026.1768761)

**Supplementary Material**


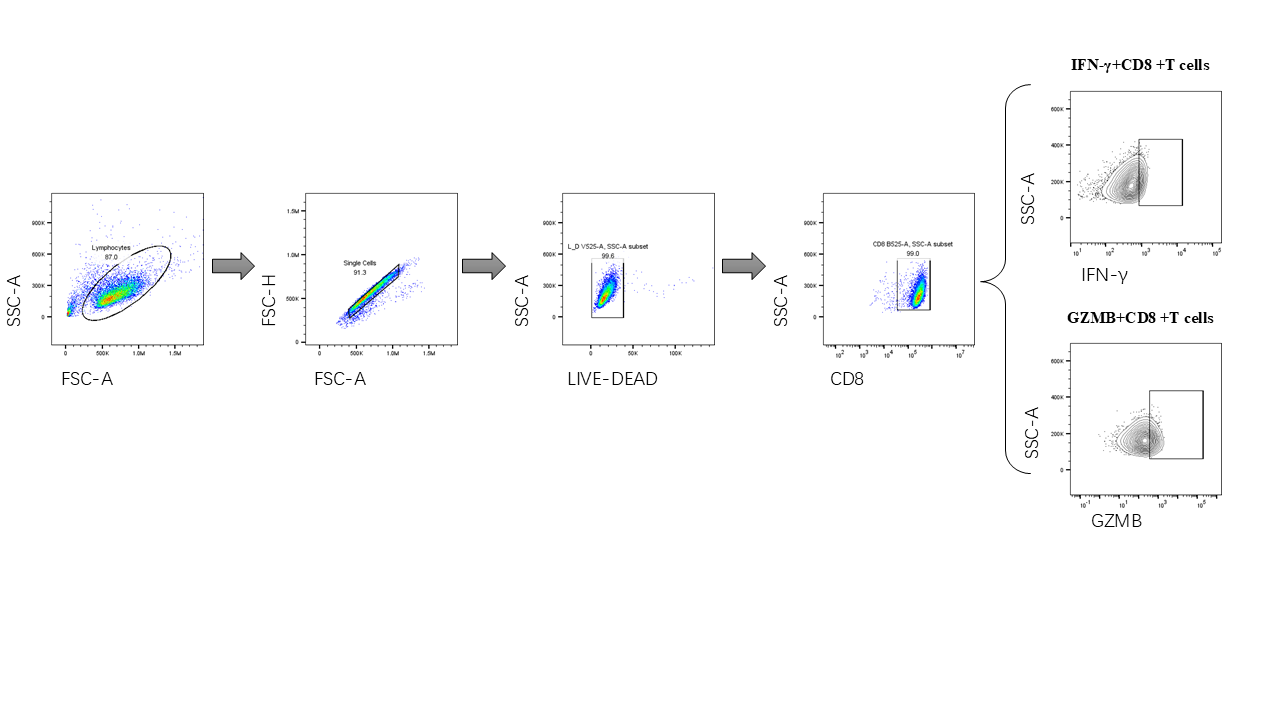


**Figure S1.** Details of gating strategies for flow cytometry.

Supplement: Supplementary file 3 [file Table3.docx]
